# A Standard Framework for Evaluating Large Health Care Data and Related Resources

**DOI:** 10.15585/mmwr.su7303a1

**Published:** 2024-05-09

**Authors:** Suad El Burai Felix, Hussain Yusuf, Matthew Ritchey, Sebastian Romano, Gonza Namulanda, Natalie Wilkins, Tegan K. Boehmer

**Affiliations:** ^1^Inform and Disseminate Division, Office of Public Health Data, Surveillance, and Technology, CDC; ^2^Division of Environmental Health Science and Practice, National Center for Environmental Health, CDC; ^3^Division of Adolescent and School Health, National Center for Chronic Disease Prevention and Public Health Promotion, CDC

## Abstract

Since 2000, the availability and use of large health care data and related resources for conducting surveillance, research, and evaluations to guide clinical and public health decision-making has increased rapidly. These trends have been related to transformations in health care information technology and public as well as private-sector efforts for collecting, compiling, and supplying large volumes of data. This growing collection of robust and often timely data has enhanced the capability to increase the knowledge base guiding clinical and public health activities and also has increased the need for effective tools to assess the attributes of these resources and identify the types of scientific questions they are best suited to address. This MMWR supplement presents a standard framework for evaluating large health care data and related resources, including constructs, criteria, and tools that investigators and evaluators can apply and adapt.

## Background and Introduction

Since 2000, the quantity of health care data available for surveillance, research, and evaluation to guide clinical and public health decision-making has increased rapidly (*1*–*3*). Major factors for this growth have been transformations in health care information technology and its use, including the increased use of electronic health records (EHRs) and electronic laboratory records; digitization of health-related information (e.g., medical imaging and medical and pharmacy claims and transactions); increased use of wearable health-related electronic devices; and the private- and public-sector efforts for collecting, compiling, and supplying large volumes of such data (*1*,*4*–*6*). As a result, numerous health care data sources contain information related to health and health care encounters for large numbers of persons. These data are drawn from various sources including EHRs; hospital and health system administrative databases; patient surveys; payee or payor claims; and laboratory, vaccination, and pharmacy information management systems. The increased availability of health care data combined with advances in data analytic capabilities have resulted in rapid increases in the use of data to guide public health and clinical practice (*5*). These upward trends in the generation, availability, and use of health care data are expected to continue (*1*,*7*), resulting in challenges to the appropriate use of data for public health surveillance and research.

To illustrate the increasing importance of large data in research and evaluation, a PubMed search was conducted for the names of selected large health care data sources in the titles and abstracts of publications, which yielded 7,919 items as of February 29, 2024 (from any date previous); the annual number of items increased from 37 in 2004 to 1,046 in 2023. The terms “MarketScan” or “IQVIA” or “Premier Healthcare Database” or “HCUP” or “Healthcare Cost and Utilization Project” were used to identify the publications. In addition, large health care data have become important in public health emergency response. For example, CDC published approximately 90 scientific articles about COVID-19 using these types of data during 2020–2022.

The increasing use of large health care data has led to ongoing efforts to standardize the data structures, definitions, and analytic approaches applied to health care data. Examples of such efforts include the Observed Medical Outcomes Partnership Common Data Model of the Observational Health Data Sciences and Informatics Clinical Data Management Working Group (https://www.ohdsi.org/data-standardization) and the Office of the National Coordinator for Health Information Technology’s United States Core Data for Interoperability standard (https://www.healthit.gov/isa/united-states-core-data-interoperability-uscdi).

Actions to guarantee the quality (i.e., how well the data are fit for the purpose; assessed often in terms of completeness, validity, accuracy, consistency, and precision), utility (i.e., how well the data can help to address research issues of importance), and usability (i.e., how easily the data can be used) of data for their intended use also are important to consider. The potential negative effect of poor data quality on the outcomes generated by use of such data has been discussed by experts in the field (*8*,*9*). A 2014 study demonstrated how improvements in a machine learning system for normalizing medical concepts in social media text were erroneous and resulted from poor data quality (*8*). Poor quality (e.g., incomplete information for key data elements, inaccuracies in the data, and nonrepresentativeness of the data) can lead to both type 1 (false positive) and type 2 (false negative) errors. In the context of health care data, such findings could be related to the distribution of diseases, risk factors for their occurrence, and the effectiveness of treatments and prevention strategies. In addition, the limitations related to the inability to easily access and use the data, uncertainty about how the data were collected and processed, and the lack of data elements to conduct sufficiently disaggregated analysis can limit the ability to address public health research questions and program information needs. To address these challenges, reports from national and international organizations and investigators involved in work related to data quality have stressed the need for developing and implementing standard methods for assessing health care data and related resources and informing users about such data and resources (*4*,*10*–*13*).

This *MMWR* supplement presents a standard framework for evaluating large health care data and related resources. Health care data refers to data about health care–related events (e.g., health care visits, prescription fills, and laboratory tests). The standard evaluation framework uses the phrase health care data and related resources (rather than health care data) to denote a compendium of data-associated elements, including the data itself, any associated electronic or cloud-based platforms or applications required to access and use the data, and other material crucial for its appropriate use (e.g., data-related trainings and documentation). In addition, in this standard evaluation framework, large data are assumed to be those that have a high volume of information (e.g., >1 terabyte of data) and potentially, a degree of complexity (e.g., data organized in multiple related tables). The purpose of this standard evaluation framework is to provide evaluators, researchers, and public health practitioners with a comprehensive set of steps and tools they can readily apply to evaluating large health care data and related resources to better understand data characteristics, strengths, limitations, and utility for various purposes. The information generated by such evaluations will enable researchers and public health practitioners to select the data and related resources that best meet their needs and enhance their ability to use and interpret the findings from these data. The evaluation constructs, criteria, and tools provided in the standard evaluation framework can be applied and adapted as needed to various types of large health care data and related resources (e.g., EHR-based data, insurance claims data, and survey data) and in various contexts within which data are evaluated (i.e., tailored to the researchers’ priorities).

## Methods

The development of the standard evaluation framework included a review of journal articles that have proposed or discussed guidelines or methods for evaluating health care–related data and principles and methods used in evaluation of surveillance systems. The review was conducted by three authors (SF, SR, HY) of the standard evaluation framework, all of whom are experienced in conducting literature reviews and evaluating large health care data. The PubMed database search used the following search terms: (data[Title]) and (“evaluation”[Title] or “evaluating”[Title] or assessment”[Title]) and (“framework”[Title] or “frameworks”[Title] or “guideline”[Title] or “guidelines”[Title] or “recommendation”[Title] or “recommendations”[Title] or “methods”[Title]). This initial search generated 759 articles as of October 3, 2022 (from any date previous). The titles and abstracts of these articles were reviewed to select those that seemed related to methods or frameworks for evaluating health care data, which resulted in the identification of 26 articles.

After review of the full texts of the 26 articles that were initially identified, six were excluded either because they were not related to health care data or did not focus on data quality. Nine additional articles were identified through a review of the reference lists of the 20 remaining articles and through subject matter knowledge of all authors of this standard evaluation framework. The final set of 29 articles (*8*,*10*,*11*,*14*–*39*) was reviewed to identify constructs, criteria, and metrics related to health care data evaluation that were proposed or used by their respective authors. A brief summary of the literature review with evaluation criteria is provided (see Findings of the Literature Review).

Established principles and methods are used in evaluations, including evaluations of surveillance systems and related data (*14**,**40**–**46*). These include engaging with interested parties during evaluations to ensure appropriate utility of the evaluation findings and conducting assessments of data completeness and representativeness to understand the quality and applicability of the data. The evaluations of large health care data need to encompass these actions because they are similarly pertinent to determining the quality of health care data and confirming the utility of the evaluation findings. Therefore, the evaluation steps, criteria, and definitions outlined in this supplement were incorporated or adapted from existing guidelines and recommendations, when applicable, or were newly developed, where needed, to form a comprehensive framework for evaluating health care data. Furthermore, health care data evaluations need to be consistent with the principles of data modernization (*45*) so that public health data and systems are up to date and account for advancements in health informatics technology and the generation and use of large data. Finally, all evaluations need to be grounded in the principles of health equity, diversity, and inclusion. On the basis of their knowledge and experience and through consultations with internal (within CDC) and external data and evaluation experts, the authors of this standard evaluation framework identified articles and reports that outlined these principles. A brief discussion of how these principles guided the standard evaluation framework development is provided (see Results).

## Results

### Findings of the Literature Review

The 29 articles reviewed provided useful information related to criteria and methods for evaluating large health care data. Multiple articles proposed frameworks or guidelines for evaluating health care–related data, often focusing on EHR data (*8*,*14*–*23*), whereas others focused primarily on a selected set of data quality criteria (e.g., completeness, validity, and representativeness) (*10*,*11*,*24*–*29*) or a particular type of data (e.g., cancer data or nutrition data) (*30*–*39*). However, none of the reviewed articles addressed the purpose of the standard evaluation framework described in this report, which was to provide a comprehensive set (capturing all or most of the potentially key attributes) of constructs, criteria, and metrics that affect decisions related to the acquisition, access, and use of various health care data and related resources for public health research and information needs. The published articles did not provide adaptable, step-by-step guidance for planning, implementing, and reporting findings from data source evaluations or suggest templates and tools. However, the articles did provide substantial information pertinent to data evaluations and would be helpful to those involved in such activities. These articles provided substantial information for the standard evaluation framework and helped to validate the constructs, questions, and metrics.

Notable articles in the review included a framework for evaluating secondary data for epidemiologic research (*16*). In that framework, the authors identified completeness of registration of persons for whom information is intended to be captured, completeness and accuracy of the data that are registered, data size, data accessibility, data usability, costs associated with data use, the format of the data, and the extent to which the data can be linked to other data as key criteria for determining the value of the data. Another study proposed terminology for data quality assessment and a framework for secondary use of EHR data (*14*). Using a harmonized crosswalk of terminology, categories, and subcategories related to data quality proposed by other authors working in this area and various subject matter experts, the authors proposed three data quality categories: 1) conformance (examining internal and external consistency and compliance in formatting, relations, and computed values), 2) completeness (examining the presence or absence of data), and 3) plausibility (examining de-duplication, temporal consistency, and consistency among values across different data elements). These criteria were assessed within the contexts of verification (focusing on consistency within the data set) and validation (assessing conformance with other data sets). Although both of these articles provide important information helpful to data evaluations, they lack broad comprehensiveness because they do not identify and describe all potential key attributes of health care data that can affect the usefulness of a data source; analytics decisions; and the development of resultant products or provide adaptable step-by-step guidance for planning, implementing, and reporting findings from data source evaluations to address specific program needs.

Another article described a proposed framework for assessing data suitability for observational studies (*17*). The authors of that article conducted a systematic literature review that examined data used in publications of population-based observational studies, a scoping review of papers focusing on the desiderata (things that are desired) of clinical databases, and a web-based survey of data users (participants identified from various organizational email lists). The authors of the article identified 16 measures and 33 submeasures that were grouped into five domains: 1) explicitness of policy and data governance, 2) relevance, 3) availability of descriptive metadata and provenance documentation, 4) usability, and 5) quality. This framework emphasized constructs and criteria beyond the more commonly recognized ones related to data quality (e.g., completeness, accuracy, and timeliness). For example, the relevance domain included measures related to the documentation describing the health care organizations and data model, the explicitness of policy and data governance domain included submeasures related to data security and privacy, and the usability domain included measures and submeasures related to how the data have been used in published literature. Measuring these attributes is important because they can substantially affect researchers’ and programs’ ability to appropriately acquire, use, and share findings from the data (*17*,*47*).

In addition, a 2014 study (*10*) presented findings from a review of 39 published articles on public health information system data quality assessments and described the study methods used to identify 49 attributes that assessed data quality (Box). The attributes most commonly assessed were completeness, accuracy, and timeliness. The study authors grouped the 49 attributes into three domains (the data collection process, the data itself, and the use of the data) and defined two broad assessment approaches or methods that were employed (objective assessments that examine the data values directly and subjective assessments that collect information from data users and stakeholders about their perceptions about the data or from data documentation) (*10*).

BOX **Attributes used to assess data quality**

Accessibility
Accuracy or positional accuracy
Comparability
Completeness
Concordance
Confidentiality or data security
Consistency or internal consistency or external consistency
Data collection method or adjustment methods or data management process or data management
Data errors or calculation errors or errors in report forms or errors resulted from data entry
Disaggregation
Ease with understanding
Granularity
Illegible handwriting
Importance
Inappropriate fields
Inconsistencies
Integrity
Invalid data
Meeting data standards
Missing data
Nonstandardization of vocabulary
Objectivity
Periodicity
Precision
Readily useableness or usability or utility
Reflecting actual sample
Relevance
Reliability
Repeatability
Representativeness
Timeliness or updatedness or currency
Transparency
Underreporting
Use of standards
Validity

**Source: **Chen H, Yu P, Hailey D, Wang N. Methods for assessing the quality of data in public health information systems: a critical review. Stud Health Technol Inform 2014;204:13–8.

### Principles of Evaluation and Program Evaluation

Although the evaluation of large health care data and related resources has its own specific context and objectives, the approach and steps to follow and standards to apply in that process can be drawn from other general guidelines for conducting evaluations. These include CDC’s Framework for Program Evaluation, which outlined a systematic approach for evaluating public health programs and program activities (*40*). The steps, from engaging with the interested parties to ensuring the use and sharing of the lessons learned, can be adapted to other evaluation endeavors. Similarly, the CDC Framework for Program Evaluation’s standards related to utility of the evaluation findings, feasibility of the evaluation activities, propriety in the conduct of the evaluation, and accuracy of the information generated are critical criteria for judging the quality of any evaluation (*40*). In addition, any evaluation activity should adhere to guiding principles for evaluators (systematic inquiry, competence, integrity, respect for persons, and common good and equity) that were established by the American Evaluation Association (*41*).

### Principles of Data Quality and Public Health Surveillance Evaluation

The practice of assessing data in terms of completeness, validity, timeliness, representativeness, and other attributes has been a staple of surveillance system and data quality assessment activities (*14*,*42*,*43*). Conceptually, these criteria also apply to determining the overall quality of large health care data and related resources. However, surveillance systems–based data and large health care data have important contextual differences that might lead to differences in how these criteria are defined and what evaluation questions ensue from them. For example, the objectives of a surveillance system often are predefined and specific (e.g., monitoring occurrence or outbreaks for selected diseases) whereas objectives related to large health care data often are broader (e.g., for epidemiologic or clinical research and public health evaluation) and not predefined. Thus, certain criteria (e.g., the timeliness and utility of the data) might be defined and assessed differently in assessments of large health care data and related resources than they are in surveillance systems evaluations. For example, a large data set based on medical claims might be structured so that updated installments of the data are available on a monthly, quarterly, or annual basis, which might be acceptable for specific research purposes but not suitable for surveillance where situational awareness in near real time is needed.

Surveillance systems data and large health care data have other important differences to consider during an evaluation of data quality. Surveillance systems data typically contain limited patient and disease information derived from a single source (e.g., laboratories and health care professionals reporting infectious disease cases to a state or local health department) whereas health care data contain extensive patient and patient care information derived from various sources (e.g., EHRs, hospital administrative data, laboratory information systems, pharmacy information systems, and provider or payor claims). Furthermore, objectives related to the use of health care data often include assessing the health status and health-related events at the individual patient level over time and across different settings, which is not feasible with most surveillance systems data. 

### Principles of Data Modernization, Evidence-Based Decision Making, Health Equity, and Patient Privacy

A framework for evaluating data and related resources also should be aligned, where applicable, with broader initiatives for modernizing and strengthening the availability and use of data for the good of the public. Such initiatives include the Federal Data Strategy (*44*) and CDC’s Data Modernization Initiative (*45*), which represent recognized principles and practices that are important for any data source. Ensuring that the objectives, methods, and outcomes of evaluation of data and related resources are consistent with broad principles, such as the Federal Data Strategy’s principles (protecting the quality and integrity of the data and validating that data are appropriate, accurate, objective, accessible, useful, understandable, and timely) will increase support for its use and the relevance of its findings. This approach also will be better achieved by having a framework that is structured to account for and assess transformations occurring in data storage (e.g., increasing use of cloud storage and semistructured data lakes), access, and analysis (e.g., using cloud-based platforms and advanced software applications) (*45*).

During the evaluations of data and related resources, an important consideration is how well the data and related resources potentially lead to generation of evidence to support public health program activities and clinical decision-making. For example, are data elements available in appropriate formats to discern the health status of and identify health outcomes among persons and assess risk factors affecting outcomes, including social determinants of health (*48*,*49*)? Public health’s mission is to protect the health and safety of all persons (e.g., https://www.cdc.gov/about/organization/mission.htm), and inherent in this mission is the principle of health equity, which calls for benefits to accrue to all persons. This principle also applies to health care data. The National Commission to Transform Public Health Data Systems, in their report with recommendations for achieving health equity–focused data systems, stated that “[to] be meaningful, data must reflect accurate and timely information about all population groups and their individual and collective capacities to experience health and well-being” (*46*). Thus, recommendations from the commission, such as for ensuring that the data have sufficient granularity to enable assessment of health status of disadvantaged population groups and for assessing gaps in data systems (e.g., lack of standard reporting of race and ethnicity data), are objectives that need to be reflected in the framework for evaluating data and related resources.

Protection of individual privacy must be a high priority in any activity related to public health and health care data. Such protections help to ensure that persons (e.g., patients) are not harmed by such activities. Thus, large health care data should abide by applicable and relevant privacy laws, regulations, and patient protection standards. The standard evaluation framework presented herein highlights the importance of protecting individual privacy and data security.

## Framework Components for Evaluating Large Health Care Data and Related Resources

On the basis of the literature review findings, existing guidelines and principles, and the authors’ experience with performing evaluations of data and related resources, the following actions, criteria, and tools are proposed as part of a comprehensive framework for evaluating large health care data and related resources. This standard evaluation framework is not meant to be prescriptive; rather, evaluators can adapt or tailor it to the context of their evaluations (e.g., the most important knowledge needs about the data and related resources and the resources available to conduct the evaluation).

### 1. Engage with Interested Parties and Define the Context and Objectives of the Data Evaluation

The evaluation should begin with engaging interested parties to define the context and objectives of the evaluation. Interested parties are persons or groups who have an interest in the evaluation and its findings (e.g., an organization or program considering accessing and using the data and related resources for a specific purpose). Examples of potential interested partners for health care data evaluations include Federal agencies, state or local health departments, universities and educational institutions, individual researchers, health care systems and the medical community, providers of the data and related resources, and private or nonprofit organizations.

The aspects of the data and related resources to be evaluated should be determined at the outset (e.g., the data or subcomponents of them, the cloud-based platforms and applications that are required for their access, and the availability of training and data use support). Also, the circumstances associated with the evaluation and purposes for it should be clearly understood. For example, are the data needed to address research needs related to a specific public health or clinical topic, is the need for data in near real time a priority, what is the organizational capacity for receiving or accessing and analyzing data, and are the data needed for public health emergency response where knowledge about the data (e.g., about data completeness and representativeness) is needed quickly? Addressing these types of questions will enable the evaluation to be optimally tailored to the constructs to focus on (i.e., assign greater relative weight to) as well as the evaluation questions and metrics and the methods and information sources to use. 

### 2. Identify the Evaluation Constructs, Questions, Metrics, and Potential Information Sources

A set of nine evaluation constructs is suggested when evaluating large health care data and related resources (Table). The constructs are 1) general attributes of the data and data systems; 2) data coverage, representativeness, and inclusion and equity; 3) data standardization and quality; 4) data period, periodicity, and recency; 5) versatility of the data; 6) utility of the data; 7) usability of the data and related resources; 8) adaptability of the data and related resources; and 9) stability of the data.

**TABLE:** A crosswalk of suggested evaluation constructs, questions, and metrics to use when evaluating large health care data and related resources*

Evaluation construct	Evaluation question	Evaluation metric
**General attributes of the data and data system (meta data):** Description of data characteristics (e.g., sources of data, types of facilities contributing data, types of patients, and period covered in the data) and the systems used to access and use the data	1a. Who collects, organizes, and provides the data and what processes are used in doing this?	Narrative description and schematic Qualitative assessment of strengths and limitations
	1b. What types of organizations or entities contribute the data?	List of organization or entity types with description (e.g., hospitals and health systems, health information exchanges, ambulatory care providers, FQHCs, insurance companies, employers, laboratories, pharmacies, and state and local health departments) Number of each type of organization
	1c. From what types of health care settings are data available?	List of health care provider, facility, or setting types from which data are provided (e.g., inpatients, emergency department, outpatient day surgery, outpatients or ambulatory care, telehealth visits, long-term care patients, and postacute care) Number of each type of setting in most recent years
	1d. What types of data are available?	List of data types (e.g., EHRs and EMRs, discharge summaries, pharmacy or laboratory information management systems, medical or pharmacy insurance claims, hospital administrative data, surveillance systems, and surveys) Number of patients, visits, or events captured in each type of data
	1e. For what time period is the data available?	List of time periods
	1f. How is patient privacy and data security ensured?	Narrative description Qualitative assessment of strengths and limitations
	1g. What are monetary costs associated with acquisition and use of the data?	List of costs
	1h. Is a data use agreement required and what are, if any, salient restrictions related to data use and information dissemination?	Narrative description
	2a. What data platforms and applications can be used or are needed to access, manipulate, and analyze the data (if applicable)?	List of the data platforms and applications with description
	2b. What salient characteristics of the data platform, including associated software applications and tools, potentially impact (positively or negatively) users’ ability to conduct data manipulation and analysis?	Narrative description Schematics or screenshots of the data platforms and applications Qualitative assessment of strengths and limitations
	2c. What is the architecture and digital volume of the data?	Description of data architecture and schema Schematics or screenshots of the data platforms and applications Size of the data sets in terms of digital volume (number of bytes) Qualitative assessment of strengths and limitations
	3. What information is available in the data (what are the data elements) and how is the data organized (e.g., what are the sub-data sets or tables and how are they relatable)?	Tabular information on sub-data sets or data tables and data elements included in each Narrative describing the relation or linkability between the tables
**Data coverage, representativeness, inclusion, and equity:** The geographic, population, provider, and health visit coverage and representativeness of the data and how well the data address principles of health equity	4a. What is the geographic coverage of the data?	Number of different types of providers (e.g., hospitals and ambulatory care providers) contributing to the data and related resources, nationally and by region, state, county, or zip code Number of different types of health care visits or events (e.g., ambulatory care, emergency department visits, hospitalizations, laboratory tests, and prescriptions filled) captured, nationally and by region, state, county, or zip code
	4b. What is the representativeness of data in terms of the population, types of health facilities and providers, types of health care visits, and other salient health care–related events captured in the data?	Percentage of different types of existing providers (e.g., hospitals and ambulatory care providers) contributing to the data and related resources, nationally and by region, state, county, or zip code Percentage of different types of incident or prevalent health care visits or events (e.g., ambulatory care, emergency department visits, hospitalizations, laboratory tests, and prescriptions filled) captured, nationally and by region, state, county, or zip code Comparative distributions of persons’ or patients’ age group, gender, payor type, and other salient characteristics between events captured in the data and incident or prevalent events in the area or facilities from which the data are derived
	4c. How well do the data enable analysis and understanding of factors affecting health equity?	List of data elements for persons’ race, ethnicity, language, rural or urban location, income, education, poverty status, disability status, sexual orientation, social vulnerability index, and other salient social determinants of health Number of persons, patients, or events by race, ethnicity, language, rural or urban location, income, education, poverty status, disability status, sexual orientation, social vulnerability index, and other salient social determinants of health, nationally and by region, state, county, or zip code Comparative distributions of persons’ or patients’ race, ethnicity, language, rural or urban location, income, education, poverty status, disability status, sexual orientation, social vulnerability index, and other salient social determinants of health between events captured in the data and incident or prevalent events in the area or facilities from which the data are derived, nationally and by region, state, county, or zip code
**Data standardization and quality:** Use of standardized data formats and the quality of the data in terms of completeness and validity	5a. To what extent do the data conform with data standardization principles and recommendations?	List of key data elements and their definitions with assessment whether they meet recommended standards (e.g., standards established by ONC, OMB, CMS, or OMOP) Brief narrative summary of data standardization
	5b. To what extent is information complete for data elements?	Frequencies for key data elements, including observations with missing data
	5c. To what extent are data values valid values?	Percentage of observations that have a valid (feasible) value for key data elements
	5d. To what extent are data values accurate values?	Percentage of observations that have accurate values for key data elements (to the extent information is available)
	5e. To what extent are data values precise values?	Percentage of data elements that contain exact quantitative values (as opposed to value ranges), where applicable
	5f. To what extent are there duplicate observations in the data?	Tabular presentation of percentage of patients with duplicate observations and average number of duplicate observations per patient, by data table
**Data period, periodicity, and recency:** Period covered by the data, frequency of data updates, and lag time in the data	6a. What is the periodicity by which updated data are made available?	Periodicity of data updates in terms of weeks, months, or years
	6b. What is the timeliness of data updates?	Lags (if any are observed) between scheduled data updates and when the data are actually updated
	6c. What is the recency of the data?	Average, minimum, and maximum difference between date of service for the health care event and when data are available for analysis
**Versatility of the data:** Ability to create unduplicated patient and visit-level observations, analyze patient data longitudinally, develop and apply specific case definitions, and link or integrate the data with other data and related resources	7. To what extent can data be linked together to form complete and unduplicated individual patient and visit or event-level information?	Percentage of observations that can be linked to form unique and unduplicated patient health care visits or events Percentage of health care visits or events for which information available in the data can be linked at the visit or event level
	8. To what extent can the data be linked together to form longitudinal patient-level information?	Proportion of observations that can be linked to form unique unduplicated patient-level information for longitudinal analysis Percentage of patients for which information available in the data can be linked at the patient level
	9. To what extent can the data be linked together or integrated with other data?	Description of how the data can be linked to other or outside data
	10. How well can specific case or event definitions (e.g., COVID-19 inpatient admission, COVID-19 disease severity levels, and COVID-19 vaccination) be formed when analyzing the data?	List of case or event definitions successfully applied by the data users when exploring and analyzing the data Narrative summary of feedback from the data users about their experience in developing and applying specific case or event definitions
**Utility of the data:** How well the data can help to address research, evaluation, and programmatic issues of importance	11a. Can the data and related resources be used successfully to address selected potential research, evaluation, and programmatic questions important to the researcher, program, or organization?	List of use cases (e.g., topics or research questions) in published and gray literature List of unpublished use cases (research, evaluation, and programmatic questions) for which data were analyzed and provided informative results
	11b. What is a broader range of important research, evaluation, and programmatic issues or questions that are important to the researcher, program, or organization that the data and related resources can potentially help to answer?	As reported by the data users: list of research, evaluation, and programmatic questions for which the data potentially can be used to effectively address
	11c. What are the potential benefits that can be gained through this data when considering other data resources that are also available to the researcher, program, or organization (e.g., does the data and related resources help address issues or questions that are not addressable or not as well addressable using other data)?	Narrative summary developed by evaluator based on feedback from the data users, other information gathered through the evaluation, and the evaluator’s own assessment
	12a. Do the data have information on charges, costs, and expenditures and how specific or itemized are these?	List of cost-related data elements, their descriptions, and sources they are derived from
	12b. What are the sources of cost-related data (e.g., information based on pre- or postadjudicated claims)?	List of cost-related data elements, their descriptions, and sources they are derived from
	12c. What is the potentially utility of the data for conducting cost-estimate, cost-effectiveness, or cost-benefit analysis?	Narrative summary developed by evaluator based on feedback from data analyst feedback, other information gathered through the evaluation, and the evaluators own assessment
	13. Can the data be used to address various potential research and evaluation issues?	Can the data be used to address the following: • Monitor health status among populations • Rapidly identify occurrence of disease and associated comorbidities and outcomes • Identify and measure risk factors for diseases and outcomes • Track diverse groups of patients along the care continuum • Track the history of disease and health status across the lifespan • Assess persons’ access to and use of health care services and the continuity of care • Assess persons’ eligibility for and use of preventive services • Assess alignment to treatment guidelines for various diseases and conditions • Assess treatment strategies and outcomes • Describe the impact of social determinants on health status and outcomes • Support the development of comprehensive population health registries • Identify early signals of emerging or novel diseases or events (e.g., symptom clusters) of high concern • Assess population-level disease burden, burden on the health care infrastructure, or both • Assess cost of care and cost-effectiveness of patient care and preventive care strategies • Assess genetic characteristics of and variations among causal pathogens • Assess biological and laboratory markers associated with disease state, severity, and outcomes • Assess persons’ health-related knowledge, attitudes, and practices
**Usability of the data and related resources:** How easily and effectively the data can be used (i.e., the capability for and level of difficulty in manipulating and analyzing the data for specific purposes using the data platform)	14a. How do salient characteristics of the data and related resources, including data platform and associated software applications and tools, potentially impact (positively or negatively) users’ ability to conduct data manipulation and analysis?	As reported by the data users: data manipulation and analysis related advantages and limitations associated with the data, data platform, and associated software applications and programming language needs
	14b. How easily are the data accessible and analyzable through the data platform (if applicable)?	As reported by the data users: activities that were conducted successfully using the platform As reported by the data users: the level of user friendliness of the platform and associated applications Descriptions of any problems encountered by the data users when accessing and using the data through the platform Summary of strengths and weaknesses and usability of the platform and associated applications reported by the data users
	15. What is the availability and quality of data-related and the data platform–related documentation, technical support, and training?	Qualitative summary of the data users’ feedback on the extent to which the documentation, trainings, technical support, and tutorial videos are useful
**Adaptability of the data and related resources:** Ability to change or adapt the data and the mechanisms for accessing and using the data for changing research, evaluation, and program needs	16. To what extent is it possible to obtain changes to the data (e.g., additions or changes to data elements included in the data sets and how data elements are defined) to meet changing analysis needs?	Narrative based on information obtained from the data supplier
	17. Can changes be made with regard to functionalities of the data platform and how data are accessed, manipulated, and analyzed (if applicable)?	Narrative based on information obtained from the data supplier
**Stability of the data:** How consistent is the availability of the overall data and data elements over time and with regard to how the data elements are formed and defined	18a. How constant are the number and types of organization, facilities, and providers providing data across the years for which data are available?	Difference over time in the number and types of organizations, facilities, and providers providing data
	18b. How constant are the data elements across the years for which data are available?	Difference over time in the data elements available in the data sets
	18c. How constant are the definitions of the data elements (including response categories) across the years for which data are available?	Difference over time in how key data elements are defined and what the respective response categories are

**Abbreviations:** CMS = Centers for Medicare & Medicaid Services; EHR = electronic health record; EMR = electronic medical record; FQHC = Federally Qualified Health Center; OMB = Office of Management and Budget; OMOP = Observational Medical Outcomes Partnership; ONC = Office of the National Coordinator for Health Information Technology.

* Potential sources of information for the evaluation indicators and metrics outlined in the crosswalk include data-related documentation, online information about the data and related resources, communication with the data provider, peer-reviewed and gray literature, feedback from previous and present users of the data and related resources, and direct analysis of the data and exploration of the data platform.

A detailed crosswalk includes the suggested evaluation questions and metrics and potential information sources (Table). The crosswalk is meant to be comprehensive and include all evaluation constructs and most of the evaluation questions and metrics that might be important to consider when evaluating large health care data and related resources. However, the crosswalk also is meant to be flexible to the specific context and objectives of an evaluation. For example, although all nine suggested evaluation constructs are important, the relative importance of each construct might differ depending on the context of the evaluation being conducted. The evaluators and interested parties will need to discuss and decide how to address and prioritize the different constructs. Similarly, considerations such as the purposes for which the data and related resources might be used, specific information needs related to the data and related resources, and time frames and resources available for the evaluation will dictate what evaluation questions and metrics are used.

A crucial factor determining how well data and related resources are evaluated is the information available to address the evaluation metrics, and thereby, the evaluation questions and constructs. This information will need to be carefully considered when identifying the metrics, questions, and constructs. Typically, three types of information sources can inform the evaluation: 1) available documentation (e.g., reports and web-based information describing the data and associated data platforms, data dictionaries, and publications and presentations resulting from the use of the data), 2) direct analysis of the data and use of associated data platforms and applications (e.g., analysis related to completeness and validity of the data), and 3) feedback from others who have used the data (e.g., previous users or pilot users of the data).

### 3. Develop Data Collection Methods and Instruments, Gather Evidence, and Analyze Data to Guide the Evaluation Metrics and Answer the Evaluation Questions

A well-structured evaluation protocol that clearly outlines the evaluation questions and metrics, what information will be collected to address the metrics, methods and tools that will be used to collect the information, and how the information will be analyzed and presented will help to facilitate implementation of the evaluation efficiently and effectively. A protocol for evaluating one or more data and related resources can be developed easily by the evaluator or evaluation team by drawing from the evaluation constructs, questions, and metrics outlined in a crosswalk (Table). These questions and metrics can be adapted, and others added, based on the context and evaluation objectives. Ideally, the evaluation protocol should clearly outline the objectives; identify the stakeholders of the evaluation; and include the evaluation questions, the metrics that will answer those questions, and the methods (including information sources) that will be used to generate those metrics.

### 4. Discuss Findings and Conclusions with Interested Parties and Support the Use of Evaluation Findings

The findings of an evaluation are only useful if they address the information needs of interested parties and if the conclusions are acceptable to them. Ensuring that the previous steps, including identification of the construct weights, evaluation questions, metrics, and the use of appropriate methods and tools in collecting data, were implemented with appropriate rigor will help to facilitate greater acceptance and use of the evaluation findings. Strengths and limitations of the data and overall conclusions about the data, in context of the needs of the interested parties, should be identified based on the evaluation’s findings. A template for a brief summary report of the findings and conclusions of the evaluation (Supplementary Appendix A, https://stacks.cdc.gov/view/cdc/151930), which can be part of a larger report resulting from the evaluation, and a scoring scheme to determine the unweighted and weighted evaluation scores for the data and related resources (Supplementary Appendix B, https://stacks.cdc.gov/view/cdc/151930) are available. The template is meant to be an adaptable and expandable tool, and a summary does not have to follow the template. The scoring scheme can be useful when summarizing, developing conclusions from, and presenting findings. 

## Practical Application of the Standard Evaluation Framework

CDC applied the standard evaluation framework, or precursors of it that guided its development, in the evaluations of multiple large health care data and related resources. These evaluations were or are being conducted as part of the mission of the CDC Data Hub program, which serves as a centralized resource for evaluating and acquiring large health care data and related resources, facilitating data access and use by CDC staff members, and providing scientific and technical support (e.g., related to understanding of data characteristics and analysis of data) to data users. Certain evaluations also were conducted to support CDC’s COVID-19 response, which required expedited identification, assessment, and use of large health care data to address priority public health research and information needs.

The standard evaluation framework was used to evaluate four large health care data and related resources that included patient-level data from health care visits in the United States; the number of patients included in each data source ranged from 7 million to 188 million. Data were derived from electronic medical records, hospital discharge and billing records, health insurance claims, and laboratory information systems. Certain salient strengths observed among these data and related resources were the capture of large numbers of patients and patient visits from all U.S. Census regions, inclusion of multiple data elements (e.g., related to patient demographics, diagnoses, procedures, laboratory test results, and visit dates) often needed in epidemiologic studies, ability to link patient information (e.g., demographics, diagnoses, and procedures) at the level of the health care encounter as well as longitudinally, and demonstrated utility of the data and related resources (e.g., multiple publications based on them). Challenges associated with the use of these data and related resources included the need for cloud-based data platforms with high-performance computing capabilities and data users’ specialized programming knowledge (e.g., SQL or PySpark) to use the data. However, such platforms, associated applications, and programming languages did enhance the potential capabilities for data manipulation and analysis. Although each data source represented millions of patients, certain of which included persons from every U.S. state, none included a statistically representative population of patients or events or the ability to apply sample weights in this regard. The standard evaluation framework was a useful tool that could be adapted easily to the evaluation of various health care data and related resources. The evaluations were able to provide standardized information about the characteristics, strengths, and limitations of the data and related resources that guided agency and program activities and decisions related to data acquisition and technical support for data use.

## Limitations

The standard framework for evaluating large health care data and related resources is subject to at least three limitations. First, the standard evaluation framework is relatively new and only has been applied in a limited number of unpublished evaluations (H Yusuf, MD, CDC, personal communication, 2023). However, the flexibility of the framework and the practical advice presented should allow for application across various health care data and related resources to generate meaningful findings. Second, for the evaluation question “Can the data be used to address various potential research and evaluation issues,” the crosswalk includes a list of issues for which health care data can be used; however, this is only a suggested list, and a user of this standard evaluation framework might need to assess the utility of data for other issues (Table). The evaluation constructs and evaluation questions, which also can be considered as evaluation criteria, presented in this standard evaluation framework are not meant to be prescriptive and can be adapted by the evaluator. Finally, the focus of the standard evaluation framework is limited to health care data, particularly data related to persons’ health care–related events. Because other types of novel data are increasingly available (e.g., mobility data and weather-related data) that can be used in public health research and surveillance, the need for knowledge about data and related resources also has increased. However, addressing such needs is beyond the scope of this standard evaluation framework and would make it unwieldy and impractical.

## Conclusion

The increasing availability of large volumes of digitized information about patients, health care–related events, and health care encounters and the technological advances that are enabling the accumulation, storage, and processing of that information will strengthen researchers’ ability to generate insights for preventing and managing diseases and protecting the population’s health. However, these advances in data and technologies also increase the challenge for ensuring that data are appropriately collected, organized, provisioned, and used. Failure to identify and use the right data for the intended purposes can result in limited value gained from investment in health care data assets. Increased scrutiny of data and the systems associated with their use through standardized evaluation approaches will help to avoid these pitfalls and influence the development of data and related resources that meet the needed standards. For example, the criteria outlined in this standard evaluation framework guide data solicitations and acquisition processes of the CDC Data Hub.

Knowledge about the characteristics and quality of large health care data and related resources, based on rigorous and standard methods, is needed and must be available to guide program decisions and use of such data. The evaluation framework described in this supplement and the associated template and tools should be helpful to those conducting evaluations of large health care data and related resources.
